# Clinical decision fatigue as a reversible state in the continuum of professional exhaustion

**DOI:** 10.3389/frhs.2026.1810631

**Published:** 2026-04-02

**Authors:** Serena Petrocchi, Luca Gabutti, Nicola Grignoli

**Affiliations:** 1 Institute of Family Medicine, Faculty of Biomedical Sciences, Università Della Svizzera Italiana, Lugano, Switzerland; 2 Cantonal Socio-Psychiatric Organisation, Public Health Division, Department of Health and Social Care, Repubblica e Cantone Ticino, Mendrisio, Switzerland

**Keywords:** burnout, compassion fatigue, medical decision-making, moral distress, motivation, decision fatigue

## Abstract

Concerns about clinician fatigue typically focus on burnout and workload, yet less attention has been paid to how the process of making repeated, emotionally and ethically challenging decisions may transiently affect decision quality. The concept of clinical decision fatigue (CDF) remains debated, partly because empirical findings differ across clinical contexts. We propose that CDF is best understood as a short-term, reversible state arising from sustained self-regulatory demands during emotionally and morally salient clinical decision-making. Drawing on contemporary process models of self-regulation, we argue that repeated exposure to empathic strain, moral conflict, and responsibility for consequential outcomes can shift clinicians’ motivational and attentional priorities away from effortful, reflective decision-making toward simplified or defensive strategies. This state is distinct from compassion fatigue and burnout in its timescale, mechanisms, and phenomenology, but may interact with these conditions when episodes recur without recovery. We argue that clinical decision fatigue is a reversible state of regulatory strain that can be situated within a broader continuum of professional exhaustion. Clinical environments that intensify emotional and moral load may accelerate the onset of CDF, whereas structures that support emotion regulation, ethical clarity, and shared decision-making may mitigate it. Recognizing CDF as a modifiable systems risk highlights opportunities to protect decisional integrity, patient safety, and clinician mental health.

## 1 Introduction

Medical decision-making requires sustained mental self-regulation. This effort may contribute to clinical decision fatigue (CDF), a process shaped by both individual characteristics and situational demands that may affect decision quality and clinician well-being. The exact nature of CDF remains debated, with mixed empirical findings across clinical contexts. The existing literature on CDF (1–4), and particularly our systematic and scoping review (2), identifies three intertwined sources of determinants: cognitive (i.e., processing complex information under stress), emotional (i.e., sustained empathic engagement with others), and moral loads (i.e., ethical conflicts and systemic constraints).

To date, there is limited empirical evidence directly supporting this emerging conceptual definition of clinical decision fatigue, partly because no validated instruments currently exist to assess it in clinical settings. Some studies have therefore operationalised decision fatigue indirectly through behavioural proxies. For example, a large-scale field study of nurses' telephone triage examined whether decision accuracy declined across sequential cases within a work shift, interpreting such deterioration as evidence of decision fatigue (5). Finding no systematic decline, the authors concluded that their data did not support the presence of decision fatigue in that context. However, this operationalisation captures only one potential manifestation of CDF—performance deterioration across repeated decisions—and may therefore not detect regulatory shifts arising from clinical encounters. The triage context examined in this study was highly protocolized, involved limited continuity of care, and minimized exposure to downstream patient consequences, conditions that may attenuate empathic and moral regulatory demands. If CDF reflects sustained emotional and ethical self-regulation, it should be less detectable in low-emotion, algorithm-supported environments and more likely to emerge in relationally embedded, high-responsibility care. Rather than refuting CDF, such findings may therefore help delineate its boundary conditions.

This is essential to be clarified for health services research to protect decisional integrity, patient safety, and clinician mental health. Our prior systematic and scoping review (2) synthesized empirical definitions, determinants, and reported manifestations of clinical decision fatigue across contexts. The present article advances a distinct contribution. A step forward in the CDF conception is necessary and we propose here a mechanistic interpretation grounded in contemporary models of self-regulation that clarify the conditions under which CDF is likely or unlikely to emerge. This article proposes a conceptual framework in which clinical decision fatigue is understood as a reversible state arising during demanding clinical decisions and situated within a broader continuum of professional exhaustion. This opinion, therefore, moves from a descriptive synthesis toward a theoretical specification of the CDF with potential implications for healthcare services.

## 2 Clinical decision fatigue as a self-regulatory process

Traditional accounts of decision fatigue have often been grounded in resource-based models of self-control. Within this framework, repeated acts of decision-making are thought to draw upon a limited pool of regulatory resources, leading over time to reduced self-control and greater reliance on heuristics or default options (6). In this view, decision fatigue emerges as the progressive depletion of an internal resource required for effortful cognitive regulation. Although highly influential, the resource-depletion model has been subject to substantial debate. Large-scale replication efforts have questioned the robustness of ego-depletion effects (7). For this reason, our framework does not assume the existence of a finite self-regulatory resource. Instead, we draw on process-based accounts that conceptualize fatigue-related changes in terms of shifts in motivational priorities and attentional allocation. Inzlicht et al. (8), for example, proposed that what appears as depletion may reflect a reallocation of cognitive priorities, in which attention and motivation gradually shift away from effortful goal-directed regulation toward states that prioritize cognitive ease or immediate reward. Experimental studies in non-clinical contexts suggest that sustained exertion of cognitive control can be followed by shifts in motivational priorities, including increased sensitivity to rewarding stimuli and reduced motivation to engage in further effortful control (8–10).

Applied to the healthcare settings, this perspective may offer a possible interpretive framework for understanding clinical decision fatigue. Rather than reflecting the exhaustion of a regulatory resource, CDF may arise from sustained engagement in demanding clinical decision-making that alters how attentional and motivational priorities are allocated over time. Direct empirical evidence demonstrating such motivational and attentional shifts in physician decision-making is currently limited. We therefore present this framework as a conceptual model that may help organize observed patterns of fatigue-related changes in clinical decision behavior and guide future empirical investigation.

### 2.1 Drifting away from empathic resonance in decision-making

Within this regulatory shift, empathy may represent a particularly vulnerable process. Effective clinical decision-making requires physicians to regulate affective resonance through cognitive perspective-taking, maintaining empathic concern while avoiding personal distress. When this regulatory balance becomes strained, patients' distress may be internalized and coupled with anxiety about causing harm or making errors. This process has been described in the literature in reference to *empathic fear* and *compassion fatigue* (11–13). At the same time, situations may arise in which clinicians recognize what they consider the ethically appropriate course of action but are unable to pursue it due to institutional or role-related constraints, giving rise to moral distress. The concept of moral residue and the crescendo effect (14) provides a potentially informative parallel. Repeated experiences of constrained ethical action may lower the threshold at which subsequent decisions trigger regulatory strain, thereby amplifying susceptibility to CDF episodes. In this sense, moral distress may function both as a precipitating factor and as an amplifier of motivational reallocation over time. Across clinical encounters, these repeated acts of self-regulation accumulate, contributing to the phenomenon of CDF. Within health service environments, such cumulative regulatory strain may alter not only clinician experience but also the consistency and defensibility of clinical judgments. We hypothesize that as this self-regulatory effort continues, motivational priorities may shift toward immediate relief, and attentional resources may become increasingly oriented toward efficiency and self-preservation rather than deliberative processing. The result would be a motivational shift: physicians may feel emotionally detached, cognitively dulled, or morally disengaged as they prioritize self-regulation. Decision quality suffers, ethical sensitivity wanes, and reliance on routine or defensive choices increases. This state is suggested to be acute, context-bound, and reversible once regulatory equilibrium is restored.

For CDF to function as a scientifically tractable construct, it must be operationalizable. We conceptualise CDF as a state-level motivational shift that emerges from sustained self-regulatory engagement in emotionally, cognitively, and morally demanding clinical decisions, leading to reduced regulatory control and increased reliance on simplified decision strategies. Clinical decision fatigue may be assessed through state-level indicators of motivational and regulatory strain, including increased perceived effort cost, reduced willingness to engage in deliberative thinking, heightened desire to disengage from decision tasks, and a preference for rapid closure or relief from continued decision-making demands. Recovery would be operationally defined as restoration of baseline decisional patterns following rest, supervisory reflection, or contextual relief. These criteria render the construct empirically testable and distinguish it from chronic distress syndromes. Future empirical studies will be necessary to determine whether these proposed indicators reliably capture regulatory strain during clinical decision-making.

### 2.2 A state-level manifestation of regulatory overload

When unmitigated, recurrent episodes of CDF may accumulate, contributing over time to the disengagement commonly observed in healthcare professional exhaustion. However, CDF is conceptually distinct from compassion fatigue and burnout in timescale, underlying mechanisms, and phenomenology, operating as a short-term, situational process that may be intertwined with these more chronic conditions. Compassion fatigue reflects a gradual, empathy-driven form of distress resulting from repeated exposure to others' suffering, characterized by reduced compassionate capacity and defensive emotional withdrawal (13, 15). Burnout is a chronic and systemic condition driven by organizational stressors and a sustained imbalance between demands and resources, characterized by pervasive emotional exhaustion and interpersonal detachment (16).

**Figure 1:**
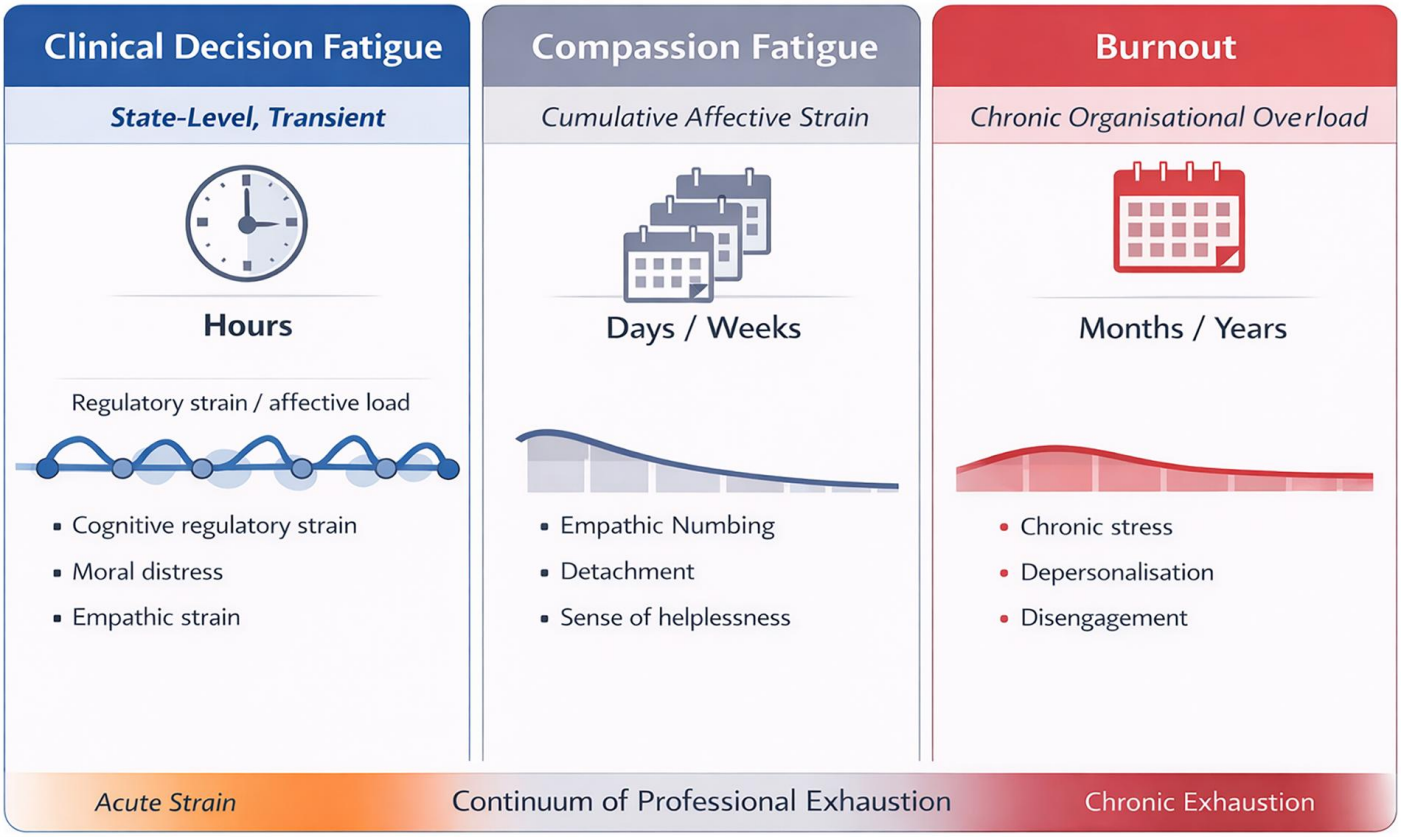
The continuum of professional exhaustion. The continuum represents a conceptual model of increasing chronicity under sustained, unresolved regulatory strain; progression is not deterministic.

CDF differs from both since it may represent a transient, decision-specific state that unfolds over hours within a shift and reflects short-term fluctuations in attentional and motivational regulation during emotionally and morally salient decision-making processes. Clinically, it may be associated with temporary indecision, increased reliance on heuristics, and momentary reductions in emotional and ethical sensitivity that resolve with recovery. Within this framework, CDF is best interpreted as a state-level manifestation of regulatory overload embedded within a broader ecology of clinician fatigue syndromes. CDF, compassion fatigue, and burnout may be conceptualised along a continuum of increasing chronicity, although movement along this continuum is neither linear nor inevitable (see Figure 1).

We propose that recurrent, unresolved episodes of CDF may represent one proximal pathway through which chronic clinician distress can emerge. However, progression is not inevitable. Whether acute regulatory strain resolves or consolidates likely depends on moderators including recovery opportunities, emotion-regulation capacity, organisational buffering, and exposure to sustained moral conflict. CDF should therefore be viewed as a potentially contributory mechanism rather than a deterministic precursor of burnout or compassion fatigue.

## 3 Discussion: system-level implications and future perspectives

Understanding CDF as a system-sensitive and potentially reversible state shifts attention from individual blame toward organisational design. If emotionally and morally demanding decisions intensify regulatory strain, institutional structures that distribute responsibility and support reflective processing may help mitigate such strain. Several organisational approaches discussed in the healthcare literature are relevant in this regard. Structured ethical debriefings following complex cases, for example, have been proposed as mechanisms for processing morally challenging experiences and reducing the accumulation of unresolved moral distress (14, 17). Similarly, shared or team-based decision-making models can distribute decisional responsibility and reduce the cognitive and moral burden placed on individual clinicians, particularly in organisational climates that support psychological safety and collaborative problem-solving (18, 19) Organizational practices that limit prolonged exposure to high-acuity environments, such as rotation systems or workload redistribution, may also reduce sustained regulatory strain associated with repeated high-stakes decisions. Supervisory and reflective frameworks, including facilitated case discussions or reflective rounds such as Schwartz Rounds, have likewise been proposed as mechanisms for supporting clinicians' emotional processing and ethical deliberation (20, 21).

Although empirical research specifically targeting clinical decision fatigue remains limited, a broader body of work links clinician distress, workload pressures, and moral strain to safety outcomes and quality of care (22). These findings support the plausibility that sustained regulatory strain may have consequences not only for individual clinicians but also for healthcare systems. From this perspective, organisational contexts that repeatedly expose clinicians to emotionally and ethically demanding decisions without adequate opportunities for recovery may contribute to shifts along the broader continuum of professional exhaustion. Framing CDF at the system level, therefore, invites prospective investigation of organisational interventions aimed at preserving decisional integrity and supporting clinicians' regulatory capacity.
